# Environmental and Occupational Exposure to Chemical Agents and Health Challenges I—What Message Can Bring to Regulatory Science?

**DOI:** 10.3390/toxics12110778

**Published:** 2024-10-25

**Authors:** Carina Ladeira

**Affiliations:** 1 H&TRC-Health & Technology Research Center, ESTeSL-Escola Superior de Tecnologia da Saúde, Instituto Politécnico de Lisboa, 1990-096 Lisbon, Portugal; carina.ladeira@estesl.ipl.pt; 2 NOVA National School of Public Health, Public Health Research Centre, Universidade NOVA de Lisboa, Comprehensive Health Research Centre (CHRC), 1600-560 Lisbon, Portugal

Human beings live in constant contact with chemical agents—mainly through environmental exposure—and also derived from the occupational settings. Environmental exposure is ubiquitous, originating from air, water, and soil [1]. While some chemicals are short-lived in the environment and may elicit no harmful effects in humans, other chemicals bioaccumulate and/or persist for a long time in the environment or the human body due to frequent exposure. However, it is valuable to refer that environmental exposure also includes diet, lifestyle, hobbies, and exposure to other substances such as drugs, food additives, pesticides, and nanomaterials, among other daily products, which are currently significant areas of research, such as bisphenols, per- and polyfluoroalkyl substances (PFAS), micro-nanoplastics (MP-NPs), among others [2,3,4,5,6,7,8]. Occupational exposure concerns the potentially harmful exposure to hazardous chemicals in the workplace; however, more specifically, it involves a substantial contact with hazardous substances [9]. Possible health effects can arise from these types of exposure, which can be measured and prevented by biomonitoring, and the outcome should be integrated to ensure better regulatory decision making.

This editorial provides a brief overview of the major findings of each of the research published, and taken together which information can give to surveillance health and regulatory science on the future research that should be considered.

This Special Issue is focused on 9 selected topics, one perspective paper (contribution 1), 2 reviews (contributions 2 and 3), and six original research papers (contributions 4–9), being three regarding human biomonitoring and occupational exposure (contributions 4-6), one in human biomonitoring and environmental exposure (contribution 7), other of environmental exposure (contribution 8), and an in vitro study (contribution 9).

The perspective paper from Myriam Borgatta and Florian Breider (contribution 1) addresses important health concerns related to inhalation of microplastics (MPs), taking in consideration three aspects—particles characteristics, additives present in plastics, and exogenous substances absorbed onto them, aiming to present a comprehensive toxicological profile of deposited MPs in the lungs, encompassing local and systemic effects.

The systematic scoping review from Ladeira et al. (contribution 2) gathered around 300 original studies of human biomonitoring of environmental and occupational exposures to chemicals using comet assay as biomarkers of DNA damage. The studies are divided in six groups of chemicals, namely: air pollutants, anaesthetics, antineoplastic drugs, heavy metals, pesticides and solvents; covering a wide range of chemicals which have been target of human biomonitoring activities.

The review from Ma et al. (contribution 3) provides an extensive overview of the application scenarios, classification, and challenges associated with hand sanitizer gels (HSGs)—due to SARS-CoV-2 their use highly increased—while emphasizing the emergence of novel components with biological functions, aiming to contribute to the advancement of hand hygiene practices and offer novel insights for the development of novel HSGs with outstanding antimicrobial properties with other multiple biological functions and desirable biosafety profiles.

Regarding the studies of human biomonitoring in occupational exposure, the study from Fustinoni et al. (contribution 4) investigated the kinetics of excretion of a new generation perfluoroalkyl surfactant (cC_6_o_4_) using blood and urine samples from workers occupationally exposed to this substance. The study concluded that cC_6_o_4_ has a shorter biopersistence in comparison with legacy per- and polyfluoroalkyl substances (PFAS), giving important scientific based evidence information which can directly impact workers health and the regulatory bodies for substances substitution. The study from Cui et al. (contribution 5) used urinary biomarkers to estimate benzene exposure levels in individuals exposed to benzene. In order to achieve that, biomarkers of internal exposure to benzene were quantified by UPLC-MS/MS such as trans, trans-muconic (*t*,*t*-MA) and S-phenyl mercapturic acid (S-PMA) metabolites, and a simple pharmacokinetic model was used to calculate back-calculated airborne benzene levels (BCABL). The results of this study showed that the *t*,*t*-MA-based BCABL can reflect the actual airborne benzene level and that the S-PMA-based BCABL is more reliable for non-professional benzene exposure. These results are very important since can offer a new approach, based on modelling, which can be use in occupational and in environmental exposures monitoring. The study from Bunsri et al. (contribution 6) explored hematological and biochemical effects of occupational pesticide exposure of Thai vegetable farmers. It was found a variety of parameters, both hematological and biochemical, to be different between pesticides users and non-users in general, and in female pesticides users in particular, concluding that the pesticide use among Thai vegetable farmers may cause hematological alterations and increase the risk of hepatic and renal dysfunction. These results showed that some hematological and biochemical parameters may be used for monitoring and surveillance in occupational exposure to pesticides.

The work from Salamanca-Fernández et al. (contribution 7) present results from phthalate metabolites and protein of brain-derived neurotrophic factor (BDNF) levels environmental exposure, among European children from five HBM4EU aligned studies and its relation with neurodevelopmental alterations. For context, the EU Horizon European Human Biomonitoring Initiative (HBM4EU) yielded data on human exposure to chemicals from harmonized human biomonitoring studies across Europe. The data provide a picture of how chemicals burden the body and impact health in Europe. For the study, it was used data from 5 cohorts from Italy, Slovakia, Slovenia, Hungary, and Norway. In brief, the results suggested that phthalates metabolites associated with more externalizing problems in boys, and a higher exposure to a non-phthalate plasticizer may associate with lower systemic BDNF levels also in boys; higher phthalate exposure is associated with higher urinary BDNF concentrations; and higher urinary BDNF concentrations may predict internalizing problems.

The work from Passarelli et al. (contribution 8) showed environmental exposure assessment results of mercury (Hg), from gold mining activity, in six provinces of the Ecuadorian Amazon region in order to assess the resulting risks to environment and human health using deterministic and probabilistic methods.

The work from Cuéllar-Pérez et al. (contribution 9) is an in vivo study, which used male Wistar rats to study the effects of blocking the brain-derived neurotrophic factor receptor TrkB on microglia. The study evidenced that BDNF and its receptor TrkB are involved in sodium cyanide (NaCN) stimulation. The TrkB receptor inhibitor, K252a, significantly inhibited microglial reactivity, indicating a role for the tyrosine-kinase receptor in these changes.

Although all papers have its major focus on Toxicology, there is a wide variety of research approaches to the published studies, from in vitro to One Health concept, and from (human and environmental) biomonitoring original researches and systematic review.

In summary, all the studies have as major objective to achieve more knowledge, at a toxicological point of view, for a better health promotion and surveillance, and disease prevention. There is an effort from the scientific community to gather information regarding the reliable value of the use of biomarkers—exposure and/or effect—in environmental and occupational exposure assessment, and to correlate or incorporate with in silico methods, such as pharmacokinetic models, allowing other the extraction of accurate information in less invasive way. The expectation is that the results taken from these studies can add data and integrate information to encourage national and international bodies to make decisions regarding old used and newly identified chemicals substances from environmental and occupational exposures; and to move forward to continuing the research needed to fill the still existing knowledge gaps.

## Data Availability

Not applicable.
